# Superficial parotidectomy versus partial superficial parotidectomy in treating benign parotid tumors

**DOI:** 10.3892/ol.2014.2743

**Published:** 2014-11-27

**Authors:** GANG HUANG, GUANGQI YAN, XINLI WEI, XIN HE

**Affiliations:** 1 Department of Oral and Maxillofacial Surgery, General Hospital of Benxi Iron and Steel Co., Benxi, Liaoning 117000, P.R. China; 2 Department of Oral and Maxillofacial Surgery, School of Stomatology, China Medical University, Shenyang, Liaoning 110002, P.R. China

**Keywords:** parotid tumor, superficial parotidectomy, partial superficial parotidectomy, quality of life

## Abstract

The aim of the present study was to compare the outcomes of superficial parotidectomy (SP) and partial superficial parotidectomy (PSP) in treating benign parotid tumors. Individuals who had undergone SP or PSP between 2005 and 2008 were enrolled, the medical records were reviewed, and a questionnaire was created and mailed to the patients. For the statistical analysis, χ^2^ and non-parametric Mann-Whitney tests were used to analyze the variables. In total, 320 patients were included in the present study. Within the PSP group, immediate facial nerve weakness occurred in six patients (7.6%), and Frey’s syndrome occurred in five (6.3%). Despite this, facial nerve function recovered fully during the follow-up, and recurrence was not identified. Within the SP group, Frey’s syndrome occurred in 38 patients (15.8%), immediate facial nerve weakness in 55 patients (22.8%) and permanent facial nerve dysfunction in two patients (0.8%). However, no recurrence was evident. In total, 216 (67.5%) patients returned the questionnaire. Those with PSP demonstrated improved scores in the domains of appearance, facial contours, facial nerve function and Frey’s syndrome. Compared with SP, PSP not only decreased the incidence of Frey’s syndrome and transient facial nerve weakness, but also improved quality of life outcomes and guaranteed a low recurrence rate.

## Introduction

Parotid tumors represent 2–3% of neoplasms affecting the head and neck, and 70–85% of those occurring in the salivary glands (1). The majority of lesions are benign and affect the superficial parotid lobe. At present, surgical excision is the most effective treatment for parotid tumors, but controversy remains with respect to the use of superficial parotidectomy (SP) (2) or partial superficial parotidectomy (PSP) (3). The primary difference between the two procedures is that the branching pattern and location of the facial nerve, rather than the size and extent of the tumor, determines the magnitude of dissection and the amount of parotid tissue that is removed in SP (4). Irrespective of the chosen technique, potential morbidities following parotidectomy, including pain, facial nerve paralysis, salivary fistulae, Frey’s syndrome and a loss of sensation, may affect quality of life (QoL) (5,6). However, successful surgery should achieve good functional results. A number of previous studies have compared the two types of parotid surgery by noting the incidence of post-operative complications (7–10), few, however, have focused on QoL. Therefore, the aim of the present study was to compare the clinical outcomes of patients following SP or PSP, with a primary focus on long-term QoL.

## Materials and methods

### Ethics

The present study was approved by the China Medical University Institutional Research Committee (Shenyang, Liaoning, China), and all participants signed an informed consent agreement.

### Patients

Eligible patients were those who had undergone an SP or PSP for a previously untreated benign parotid tumor at the Department of Oral Maxillofacial Head and Neck Tumor Center, China Medical University, between 2005 and 2008. In addition, participants had to meet the following criteria in order to be included in the study: An age of ≥18 years at diagnosis, absence of any communication impairments, no post-operative radiotherapy and a period of at least five years since surgery. A QoL questionnaire was devised according to the Hebrew version of the University of Washington Quality of Life instrument (5,6) and subsequently mailed to the participants. The answers were scored according to the associated instructions, with high and low scores meaning low and high levels of complaint, respectively.

### Surgical technique

For the SP technique, the main facial nerve trunk was identified and traced to its branches. The parotid duct was then legated in all cases. Subsequently, the entire superficial parotid tissue region was excised with the tumor en bloc.

For the PSP technique, only the branches surrounding the tumor were exposed, and the parotid tumor was excised with a surrounding 0.5 to 1-cm cuff of normal parotid tissue. The parotid duct was preserved in all patients. This method removed only the tumor-bearing area, and avoided the requirement for more extensive facial nerve dissection.

### Statistical analysis

The χ^2^ test was used to analyze general variables, including age, gender and pathological diagnosis. A non-parametric Mann-Whitney test was used to compare the QoL outcomes between the two groups. Statistical analysis was conducted using SPSS version 13.0 software (SPSS, Inc., Chicago, IL, USA). P<0.05 was used to indicate a statistically significant difference.

## Results

### Patients and postoperative complications

The 320 patients enrolled in the present study were divided into two groups according to the type of parotidectomy that had been performed. The mean age of the patients in the PSP group (n=79) was 50.3 years (range, 20–74 years), and the ratio of males to females was 32:47. The pathological diagnoses were distributed as follows: 57 cases of pleomorphic adenoma (PA), 12 cases of Warthin’s tumors, six cases of basal cell adenoma (BCA) and four cases of myoepithelioma. Immediate facial nerve weakness occurred in six patients (7.6%). Despite this, facial nerve function recovered fully during follow-up, and recurrence of a parotid lesion was not identified. Frey’s syndrome occurred in five (6.3%) of the patients. The mean age of the patients in the SP group (n=241) was 52.6 years (range, 19–83 years), and the ratio of males to females was 98:143. The pathological diagnoses were distributed as follows: 170 cases of PA, 38 cases of Warthin’s tumors, 20 cases of BCA, nine cases of myoepithelioma and four cases of cystadenoma. Immediate facial nerve weakness occurred in 55 patients (22.8%), and two patients (0.8%) developed permanent facial nerve dysfunction. Recurrence of a parotid lesion was not identified. Frey’s syndrome occurred in 38 patients (15.8%) (Table I).

### Quality of life

In total, 216 patients (67.5%) returned the questionnaire. Overall, 60 had undergone PSP and 156 had undergone SP. No significant differences regarding age, gender or pathological diagnosis were identified between the groups. The mean general health score of the patients who had undergone PSP or SP was 3.47 [standard deviation (SD), 0.63] and 3.36 (SD, 0.62), respectively; the difference between groups was not significant (P=0.297). The majority of patients in each group stated that their health was now the same as it was one year prior to diagnosis. The mean overall health scores for the PSP and SP groups were 3.27 (SD, 0.58) and 3.31 (SD, 0.61), respectively; the difference between groups was not significant (P=0.733). The pain domain was scored the highest for each of the groups, with no patients reporting symptoms of pain. In the PSP group, there were no reports of negative changes in physical appearance, but 10 patients (12.7%) in the SP group reported that they were unsatisfied with the cosmetic results post-surgery. Consequently, the mean appearance score was higher in patients who had undergone PSP rather than SP (mean score, 96.7 vs. 87.8; P=0.015). The majority of patients in each group stated that the scar was barely noticeable, with mean scores of 98.33 (SD, 6.34) and 95.83 (SD, 9.38) for the PSP and SP groups, respectively; the difference between groups was not significant (P=0.180). The mean scores for facial contour changes were 96.67 (SD, 8.64) for the PSP group and 89.42 (SD, 16.86) for the SP group. Furthermore, patients who had undergone PSP were more satisfied with the outcome (P=0.038). The mean sensation domain score of patients from the PSP group was 94.17 (SD, 12.6), which was comparable with that from the SP group (91.03; SD, 14.51). The mean local effect (Frey’s syndrome) score was 97.50 (SD, 10.06) and 88.46 (SD, 21.95) for the PSP and SP groups, respectively. A larger proportion of patients who had undergone SP reported this complication (P=0.027). The fistula domain demonstrated improved scores from patients in the PSP group compared with the SP group. However, this difference was not identified to be significant (92.07 vs. 83.56; P=0.130). The mean facial function score was 97.73 (SD, 8.63) for the PSP group and 87.37 (SD, 17.38) for the SP group; this finding was revealed to be significant (P=0.002) (Table II).

## Discussion

Studies by Erkan *et al* (5) and Nitzan *et al* (6) reported that, following a parotidectomy, the general health status of the majority of patients was good. Although these studies concluded that parotidectomy did not appear to severely affect the overall QoL, they did not evaluate whether there was a difference in the QoL following different resection types. In the present study, it was also observed that the majority of patients within the total sample were in good general health, and that PSP conferred a similar general health result compared with SP.

Foghsgaard *et al* (11) examined immediate post-operative pain following SP, and identified no significant difference in the pain score between patients with a preserved or transected posterior branch of the great auricular nerve (GAN). It is likely that the reported pains were a result of surgical trauma, and therefore, whether or not the different surgical techniques had a negative effect upon pain would require further questioning subsequent to a longer follow-up period. Wormald *et al* (12) assessed pain following parotidectomy using a visual analogue scale and identified that pain appeared to have little impact upon the patients. However, the study did report one patient who presented with severe and persistent discomfort. A potential explanation for this may have been neuroma formation following damage to the GAN. In the present study, attempts were made to preserve the GAN posterior branch in each patient. As there were no reports of neuroma formation during the follow-up, patients from each of the groups demonstrated high pain scores. Furthermore, the difference in these scores was not identified to be significant between the groups.

In a study by Nitzan *et al* (6), 70% of patients reported a change in appearance; 60% due to scarring and 58% due to local depression. Therefore, it is possible that the domains of appearance, scarring and facial contours are associated, and should therefore be analyzed together. A study by Koch *et al* (7) identified that the majority of patients were not fully satisfied with the cosmetic result following surgery. However, the mean score was relatively high and the authors did not demonstrate a significant correlation between cosmetic appearance and the extent of surgery (PSP vs. SP). In the present study, it was revealed that the majority of patients who were satisfied with the cosmetic result, and those who’s mean score was significantly different to that of the SP group, were those who had undergone PSP. It was therefore hypothesized that a larger amount of gland tissue removal resulted in increased cosmetic morbidity. The findings of Ciuman *et al* (10) and Roh *et al* (13) also support this result. The majority of the parotidectomies in the present study were performed using modified Blair incisions. Previous studies (12) have suggested that this incision would result in a prominent scar, however, the majority of patients in the present study reported that the scar was barely noticeable. This may be the result of the sutures being performed by an experienced surgeon, or the post-operative application of a scar formation inhibitor.

Loss of sensation is a complication reported following GAN sacrifice. In a study by Koch *et al* (7), the mean score for loss of sensation was 4.3, with no significant difference identified between PSP and SP. This finding was consistent with that of the present study. By contrast, Ciuman *et al* (10) demonstrated statistically significant differences between sensory impairment and the extent of surgery. The duration of sensory impairment was significantly longer following SP compared with PSP. However, this finding may be the result of varied follow-up times and the possibility that sensory deficits could decrease with time (7).

Following a parotidectomy, Frey’s syndrome has been demonstrated to affect between 1.5 and 27.7% of patients (1,4,14). The pathogenesis is believed to be the aberrant healing of sympathetic and parasympathetic nerves. In a study by Emodi *et al* (4), Frey’s syndrome occurred in 27.7% of the patients: Four cases treated with SP and nine cases treated with PSP. Similarly, Papadogeorgakis *et al* (15) reported that Frey’s syndrome affected 18% of patients who had undergone SP, and 5% who had undergone PSP. However, these studies did not reveal a significant difference in the incidence of Frey’s syndrome in patients treated by PSP or SP, which may have been the result of small sample sizes. The present study, which consisted of 320 patients, identified that Frey’s syndrome occurred more frequently in patients who had undergone SP (P=0.033). This may be due to the fact that less parotid tissue was removed by SP, and as the parotid fascia was closed directly (15,16). Furthermore, in the QoL analysis, a greater proportion of patients treated by SP reported this complication. Therefore, PSP may not only decrease the incidence of Frey’s syndrome, but also achieve good QoL outcomes (10).

Permanent facial dysfunction, despite a low occurrence, was the most serious morbidity reported following parotidectomy in the present study. By contrast, immediate facial weakness was relatively common. Zhang *et al* (8) reported an overall transient palsy rate of 24%, with a lower incidence in patients who had undergone PSP compared with SP. Similarly, Witt (17) performed a 20-year review of the literature, and revealed that the incidence of transient facial nerve dysfunction was 26% for SP and 18% for PSP. In the present study, a higher rate of immediate facial weakness in patients treated by SP was also noted. This could be explained by the fact that, during PSP, less of the parotid tissue was sacrificed, and only the branches around the tumor were dissected. In the QoL analysis, it was noted that a higher number of patients who had undergone SP were affected by facial nerve paralysis. Similarly, Ciuman *et al* (10) concluded that the esthetic outcome was significantly associated with the extent of surgery, and that conventional SP was therefore more likely to result in a poorer perception of appearance. Furthermore, no significant difference regarding the recurrence rate in patients who had undergone either PSP or SP was revealed. Previous studies have also reported no increase in the recurrence rate following limited parotid resection (4,10,15,16,18). Lim *et al* (19) even described the clinical reliability of conservative parotidectomy for the treatment of parotid cancers.

In summary, compared with SP, PSP not only has the potential to decrease the incidence of Frey’s syndrome and transient facial nerve weakness, but can also result in improved QoL outcomes and lower recurrence rates.

## Figures and Tables

**Table I:** Analysis of patient characteristics in the PSP (n=79) and SP (n=241) groups.

Variables	PSP group	SP group	P-value^a^
Mean age (range), years	50.3 (20–74)	52.6 (19–83)	>0.05
Gender, n			
Male	32	98	>0.05
Female	47	143	
Diagnosis, n			
Pleomorphic adenoma	57	170	>0.05
Warthin’s tumor	12	38	
Basel cell adenoma	6	20	
Myoepithelioma	4	9	
Cystadenoma	0	4	
Immediate facial paralysis, n	6	55	0.003
Permanent facial dysfunction, n	0	2	>0.05
Frey’s syndrome, n	5	38	0.033
Recurrence, n	0	0	>0.05

a χ^2^ test. PSP, partial superficial parotidectomy; SP, superficial parotidectomy.

**Table II:** Quality of life comparison between patients treated by PSP and SP.

Domain	PSP group	SP group	P-value
General health	3.47	3.36	0.297
General health compared with 1 year prior to diagnosis	3.27	3.31	0.733
Pain	100.00	100.00	1.000
Appearance	96.68	87.80	0.015
Scar	98.33	95.83	0.180
Facial contours	96.67	89.42	0.038
Sensation	94.17	91.03	0.360
Frey’s syndrome	97.50	88.46	0.027
Facial nerve function	97.73	81.37	0.002
Fistula	92.07	83.56	0.130
Mouth dryness	2	4	0.639
Dryness contributed by surgery	0	2	0.467

PSP, partial superficial parotidectomy; SP, superficial parotidectomy.

## References

[1] Upton DC, McNamar JP, Connor NP, Harari PM, Harting GK (2007). Parotidectomy: ten-year review of 237 cases at a single institution. Otolaryngol Head Neck Surg.

[2] Pia F, Policarpo M, Dosdegani R, Olina M, Brovelli F, Aluffi P (2003). Centripetal approach to the facial nerve in parotid surgery: personal experience. Acta Otorhinolaryngol Ital.

[3] Bhattacharyya N, Richardson ME, Gugino LD (2004). An objective assessment of the advantages of retrograde parotidectomy. Otolaryngol Head Neck Surg.

[4] Emodi O, El-Naaj IA, Gordin A, Akrish S, Peled M (2010). Superficial parotidectomy versus retrograde partial superficial parotidectomy in treating benign salivary gland tumor (pleomorphic adenoma). J Oral Maxillofac Surg.

[5] Erkan AN, Yavuz H, Ozer C, Ozer F, Ozluoglu L (2008). Quality of life after surgery for benign disease of the parotid gland. J Laryngol Otol.

[6] Nitzan D, Kronenberg J, Horowitz Z (2004). Quality of life following parotidectomy for malignant and benign disease. Plast Reconstr Surg.

[7] Koch M, Zenk J, Iro H (2010). Long-term results of morbidity after parotid gland surgery in benign disease. Laryngoscope.

[8] Zhang SS, Ma DQ, Guo CB, Huang MX, Peng X, Yu GY (2013). Conservation of salivary secretion and facial nerve function in partial superficial parotidectomy. Int J Oral Maxillofac Surg.

[9] Dell’Aversana Orabona G, Bonavolontà P, Iaconetta G, Forte R, Califano L (2013). Surgical management of benign tumors of the parotid gland: extracapsular dissection versus superficial parotidectomy - our experience in 232 cases. J Oral Maxilllofac Surg.

[10] Ciuman RR, Oels W, Jaussi R, Dost P (2012). Outcome, general, and symptom-specific quality of life after various types of parotid resection. Laryngoscope.

[11] Foghsgaard S, Foghsgaard J, Homøe P (2007). Early post-operative morbidity after superficial parotidectomy: a prospective study concerning pain and resumption of normal activity. Clin Otolaryngol.

[12] Wormald R, Donnelly M, Timon C (2009). ‘Minor’ morbidity after parotid surgery via the modified Blair incision. J Plast Reconstr Aesthet Surg.

[13] Roh JL, Kim HS, Park CI (2007). Randomized clinical trial comparing partial parotidectomy versus superficial or total parotidectomy. Br J Surg.

[14] Ali NS, Nawaz A, Rajput S, Ikram M (2010). Parotidectomy: a review of 112 patients treated at a teaching hospital in Pakistan. Asian Pacific J Cancer Prev.

[15] Papadogeorgakis N (2011). Partial superficial parotidectomy as the method of choice for treating pleomorphic adenomas of the parotid gland. Br J Oral Maxillofac Surg.

[16] Papadogeorgakis N, Skouteris CA, Mylonas AI, Angelopoulos AP (2004). Superficial parotidectomy: technique modifications based on tumor characteristics. J Craniomaxillofac Surg.

[17] Witt RL (2002). The significance of the margin in parotid surgery for pleomorphic adenoma. Laryngoscope.

[18] Zbären P, Vander Poorten V, Witt RL (2013). Pleomorphic adenoma of the parotid: formal parotidectomy or limited surgery?. Am J Surg.

[19] Lim YC, Lee SY, Kim K (2005). Conservative parotidectomy for the treatment of parotid cancers. Oral Oncol.

